# Calcium Phosphate Nanoparticles Functionalized with a Cardio-Specific Peptide

**DOI:** 10.3390/nano15020094

**Published:** 2025-01-09

**Authors:** Federica Mancini, Lorenzo Degli Esposti, Alessio Adamiano, Jessica Modica, Daniele Catalucci, Dora Mehn, Otmar Geiss, Michele Iafisco

**Affiliations:** 1 Institute of Science, Technology and Sustainability for Ceramics (ISSMC), National Research Council (CNR), 48018 Faenza, Italy; federica.mancini@issmc.cnr.it (F.M.); lorenzo.degliesposti@issmc.cnr.it (L.D.E.); alessio.adamiano@issmc.cnr.it (A.A.); 2 IRCCS Humanitas Research Hospital, Humanitas Cardio Center, 20089 Rozzano, Italy; jessica.modica@hunimed.eu (J.M.); daniele.catalucci@cnr.it (D.C.); 3 Institute of Genetic and Biomedical Research (IRGB)—Milan Unit, National Research Council (CNR), 20090 Milan, Italy; 4 European Commission, Joint Research Center (JRC), 21027 Ispra, Italy; dora.mehn@ec.europa.eu (D.M.); otmar.geiss@ec.europa.eu (O.G.)

**Keywords:** cardiovascular diseases, calcium phosphate, nanoparticles, therapeutic peptides, drug delivery

## Abstract

Cardiovascular diseases (CVDs) remain the leading cause of mortality worldwide, highliting the urgent need for new therapeutic strategies. Peptide-based therapies have demonstrated significant potential for treating CVDs; however, their clinical application is hindered by their limited stability in physiological fluids. To overcome this challenge, an effective drug delivery system is essential to protect and efficiently transport peptides to their intended targets. This study introduces two distinct strategies for loading a cardio-specific mimetic peptide (MP), previously designed to modulate L-type calcium channel function in cardiomyocytes, onto calcium phosphate nanoparticles (CaP NPs). MP-loaded CaP NPs were prepared by two different wet precipitation syntheses, one of which involved the use of sodium polyacrylate as a templating agent. Characterization of MP-loaded CaP NPs showed that their crystallinity, size, surface charge, and morphology could be tuned by adjusting the synthesis parameters. In vitro tests on cardiac cells confirmed that both types of MP-loaded CaP NPs are biocompatible with HL-1 cardiomyocytes and restored intracellular calcium flux under stressed conditions, highlighting their therapeutic potential. These results pave the way for further optimization of CaP NP formulations and suggest their potential as a viable nanomaterial for CVD treatment.

## 1. Introduction

Cardiovascular diseases (CVDs) encompass a range of disorders affecting the heart and blood vessels, including coronary heart disease, stroke, and rheumatic heart disease [1,2], which collectively result in about 18 million deaths worldwide each year [3,4,5]. Current treatments primarily manage symptoms or slow disease progression [2], underscoring the need for therapies that address the underlying disease mechanisms.

Peptide-based therapies have shown great potential in treating CVDs, as they selectively target molecular pathways involved in myocardial contraction and cardiac function regulation [6,7,8]. Among the cardio-specific peptides [9,10,11,12,13], Rusconi et al. [14] developed an 11-amino acid mimetic peptide (MP) that targets the Ca_v_β2 cytosolic subunit of L-type calcium channels (LTCCs), restoring cardiac function in diseased cardiomyocites by modulating LTCC levels at the plasma membrane [8,14,15]. Despite their promise, therapeutic peptides face significant challenges, including rapid degradation by circulating enzymes, which limits their half-life in the bloodstream, and poor cellular permeation, thereby reducing their efficacy. A suitable carrier is therefore essential to protect and ensure their delivery to intracellular targets [16]. Nanoparticles (NPs) offer a promising solution for cardio-specific peptide delivery, acting as protective carriers that enhance the stability and bioavailability of peptides, while also enabling targeted delivery to specific cells or organs [8,17].

Calcium phosphate nanoparticles (CaP NPs) have recently shown substantial promise for treating CVDs by facilitating the delivery of therapeutic peptides to the intracellular space of cardiomyocytes [8,17]. Known for their biocompatibility and pH-dependent degradability, CaP NPs are widely used in biomedical applications partly due to their close resemblance to the mineral phase of human hard tissues, which helps to avoid adverse immune responses [18,19]. In addition, CaP NPs were proved to be well tolerated by cardiac cells, as demonstrated in a previous work, in which the HL-1 cardiac cell line and primary adult cardiomyocytes exposed to CaP NPs showed no alterations in terms of electrophysiology, contractility, or intracellular calcium transients [17]. Similarly, no adverse events were reported in a dose-ranging study conducted in rats exposed to incremental doses of CaP NP formulations [8].

In a recent study [8], we demonstrated that the inhalation of CaP NPs functionalized with MP enhables rapid NP translocation from the pulmonary system into the bloodstream, and subsequently via the pulmonary vein to the myocardium, where the therapeutic cargo is released into cardiomyocytes. In vivo studies on rodent models of diabetic cardiomyopathy showed that inhaled MP-loaded CaP NPs result in targeted cardiac delivery and the functional recovery of myocardial contractile capacity. Alogna et al. [17] further confirmed these findings by administering dry powders containing MP-loaded CaP NPs embedded in mannitol microparticles to minipigs with heart failure with reduced ejection fraction (HFrEF), yelding promising results.

In the above-cited studies, MP-loaded CaP NPs were synthesized using a wet precipitation approach. In detail, calcium and phosphate precursors were combined in an aqueous solution with sodium citrate, followed by a brief inubation at 37 °C for 5 min. MP was added to the phosphate precursor to achieve drug conjugation, and the resulting suspension was purified by dialysis. This approach yielded CaP NPs with a size of approximately 50–100 nm and a negative surface charge due to the presence of citrate.

In this work, we developed two novel CaP NP-based delivery systems to facilitate the loading of biologics onto these inorganic NPs. The well-established therapeutic peptide MP was used as a reference drug. By adjusting the synthesis parameters, such as the type of precursors, temperature, and reaction time, we tailored the physico-chemical properties of CaP NPs. Our aim was to establish new synthetic approaches for loading MP onto CaP NPs, thus expanding the range of MP-functionalized CaP NPs for various therapeutic applications in CVDs. Notably, the fate of NPs when admistered in vivo, including via inhalation, largely depends on their size, shape, and surface charge. Initially, we synthesized MP-loaded CaP NPs without the use of template molecules. In the subsequent phase, polymer-functionalised MP CaP NPs (PAA MP CaP NPs) were prepared using sodium polyacrylate (PAANa) as the template. PAANa is an analogue of polyaspartic acid, which is believed to promote CaP biomineralization of human hard tissues [20]. The physico-chemical properties of MP CaP NPs and PAA MP CaP NPs were thoroughly characterized, and the in vitro biocompatibility with HL-1 cardiomyocytes cells, as well as their functional effectiveness in restoring cardiac function under stressed conditions, was studied.

## 2. Materials and Methods

### 2.1. Materials

Acetic acid (CH_3_COOH, ≥99.7% pure), acetonitrile (CH_3_CN, ≥99.9% pure), calcium acetate monohydrate (Ca(CH_3_COO)_2_∙H_2_O, ≥99.0% pure), calcium chloride (CaCl_2_, ≥99.0% pure), di-ammonium hydrogen phosphate ((NH_4_)_2_HPO_4_, ≥99.0% pure), 4-(2-hydroxyethyl)-1-piperazineethanesulfonic acid (HEPES) (C_8_H_18_N_2_O_4_S, ≥99.0% pure), hydrochloric acid (HCl, 37% pure), magnesium chloride hexahydrate (MgCl_2_∙6H_2_O, ≥99.0% pure), nitric acid (HNO_3_; 65% pure), polyacrylic acid sodium salt (PAANa, average Mw 5100), potassium chloride (KCl ≥ 99.5% pure), sodium acetate (CH_3_COONa, ≥99.0% pure), sodium carbonate monobasic (NaHCO_3_, ≥99.7% pure), sodium citrate tribasic dihydrate (Na_3_(C_6_H_5_O_7_)∙2H_2_O, ≥99.0% pure—named hereafter Na_3_Cit), sodium chloride (NaCl ≥ 99.5% pure), sodium hydroxide (NaOH, ≥98.0% pure), sodium phosphate dibasic (Na_2_HPO_4_, ≥99.0% pure), sodium sulphate (Na_2_SO_4_, ≥99.0% pure), and trifluoroacetic acid (C_2_HF_3_O_2_, ≥99.% pure—named hereafter TFA) were purchased from Sigma Aldrich (St. Luis, MO, USA) and used without further purification. The HL-1 Cardiac Muscle Cell Line and Claycomb medium were purchased from Sigma Aldrich. Opti-MEM medium was purchased from Thermo Fisher Scientific (Waltham, MA, USA). Thapsigargin (C_34_H_50_O_12_, ≥97% pure) and Ryanodine (C_25_H_35_NO_9_, ≥98% pure) were purchased from Bio-Techne (Minneapolis, MN, USA). Bay K8644 (C_16_H_15_F_3_N_2_O_4_, ≥98% pure) was purchased from Merck Millipore (Burlington, MA, USA). All products were used without further purification.

MP (sequence: DQRPDREAPRS) was purchased from GenScript (Piscataway, NJ, USA) and used without further purification.

All the solutions were prepared with ultrapure water (18.2 MΩ × cm, 25 °C, arium© pro, Sartorius, Gottingen, Germany).

### 2.2. Preparation of Nanoparticles

#### 2.2.1. Preparation of MP CaP NPs

MP CaP NPs were prepared by a wet coprecipitation method. A 20 mL aqueous solution of 0.015 M Ca(CH_3_COO)_2_∙H_2_O also containing MP at nominal concentrations of 100, 200, and 400 ppm was prepared and stirred for 30 min at room temperature. Subsequently, a 20 mL solution of 0.009 M (NH_4_)_2_HPO_4_ was added. The final mixture was introduced in a round-bottom flask, sealed with a stopper, and then heated at 60 °C for 3 h under magnetic stirring. Afterwards, MP CaP NPs particles were separated from the supernatant by centrifugation (6000 rpm, 10 min, 4 °C) and washed once with ultrapure water. The resultant pellet was resuspended in ultrapure water and stored at 4 °C. MP-free CaP NPs were prepared as a negative control following the same procedure, but without adding the peptide. An aliquot of each sample was freeze-dried at −50 °C under a vacuum of 3 mbar to perform the phyisico-chemical characterization of the NPs.

#### 2.2.2. Preparation of PAA MP CaP NPs

PAA MP CaP NPs were prepared according to a modified procedure taken from Jang et al. [21]. A 20 mL aqueous solution of 0.015 M Ca(CH_3_COO)_2_∙H_2_O containing MP at nominal concentration of 400 ppm was prepared and stirred for 30 min at room temperature. Subsequently, a 10 mL solution of 0.009 M (NH_4_)_2_HPO_4_ and 0.75 mg/mL PAANa (10 mL) were added to the mixture. The final mixture was then introduced in a round-bottom flask, sealed with a stopper, and then heated at 50 °C for 1 h under magnetic stirring. After precipitation, the particles were separated from the supernatant by centrifugation (7000 rpm, 10 min, 4 °C) and washed once with ultrapure water. The resultant material was resuspended in ultrapure water and stored at 4 °C. Negative controls, i.e., MP-free PAA CaP NPs and PAA-free MP CaP NPs formulations, were prepared following the same procedure, but without adding MP or PAANa solution to the mixture. An aliquot of each sample was freeze-dried at −50 °C under a vacuum of 3 mbar to perform the physico-chemical characterization of the produced NPs.

### 2.3. MP Quantification by HPLC

Quantification of MP was carried out by High-Performance Liquid Chromatography (HPLC) using a 1260 Infinity II LC System (Agilent Technologies, Santa Clara, CA, USA). Separation was obtained using an Agilent Eclipse Plus C18 column (250 mm × 4.6 mm, 5 μm, Agilent Technologies, Santa Clara, CA, USA). The mobile phase was composed of an aqueous TFA solution (0.065% *v*/*v*) and acetonitrile. Samples were eluted at a flow rate of 1.0 mL min^−1^, the temperature was set at 25 °C, and the wavelength for MP detection was fixed at 220 nm (bandwidth 4 nm). The elution time of MP was 6.3 min. The linearity of the method was assessed in the range from 0.005 mg mL^−1^ to 0.1 mg mL^−1^ of MP standard solutions, diluted 1:1 with HCl 0.1 M solution.

### 2.4. Analytical Ultracentrifugation

Analytical ultracentrifuge (AUC) measurements were performed at 20 °C using a Proteomelab XL-I analytical Beckman Coulter ultracentrifuge (Brea, CA, USA) in an 8-hole Ti rotor, in 2-sector centrepiece cells equipped with sapphire windows using interference optics. The reference cell was loaded with 400 µL of water, while sample cells contained 390 µL of MP aqueous standard solutions or the sample (in triplicate). After 20 h at 40,000 rpm, the centrifuge was stopped, and a 200 µL aliquot of the liquid phase (supernatant) was taken from the sample sector. AUC data analysis was run using the software SEDFIT version 16.1c [22], applying the continuous c(s) distribution model in the 0.001–2 S range at 200 resolution with logarithmic s binning, considering the default density (1 g/mL) and viscosity (0.00102 mPas) values set in the software for water as solvent at 20 °C and the partial specific volume for protein (0.73 mL/g) at a fixed 1.3 frictional coefficient ratio. Peptide concentrations in the supernatant aliquots were determined with an Agilent 1290 HPLC system, composed of a high-speed pump (G7120A), a temperature-controlled multisampler (G7167B), a column oven (G7116B), and a diode array detector (G7115A). The measurement wavelength was set to 220 nm (bandwidth 4 nm) and the reference wavelength to 360 nm (reference bandwidth 100 nm). The chromatographic column contained a polar-embedded C18 phase (Phenomenex, Synergi Fusion-RP, 150 × 4.6mm, particle size 4 µm, Part No 00F-4424-E0). The flow was set at 0.9 mL min^−1^. The injection volume was 20 µL. For the mobile phase, 0.1% trifluoroacetic acid [A] and acetonitrile [B] were used and samples were diluted 1:1 with 0.1 M HCl solution prior to injection, as described above.

### 2.5. NPs Characterization

Freeze-dried CaP NPs were characterized by powder X-ray diffraction (PXRD), Fourier-transform IR spectroscopy (FT-IR), scanning electron microscopy (SEM), and thermogravimetric analysis (TGA). PXRD patterns were collected with a D8 Advance diffractometer (Bruker, Karlsruhe, Germany). Cu Kα X-rays were generated at 40 kV and 40 mA. The pattern was collected in the 10–60° 2θ range with a step size of 0.02 degrees and a counting time of 0.5 s. FT-IR spectra were collected in attenuated total reflectance (ATR) mode with a Nicolet iS5 spectrometer (Thermo Fisher Scientific Inc., Waltham, MA, USA) using an iD7 diamond ATR accessory. The spectra were collected with a resolution of 4 cm^−1^ by the accumulation of 16 scans covering the 4000 to 400 cm^−1^ spectral range. SEM micrographs of the samples were collected with a field-emission gun (FEG-SEM) ZEISS ΣIGMA microscope (ZEISS NTS GmbH, Oberkochen, Germany) with in-lens acquisition mode, operating at a 3 kV acceleration voltage, with a working distance of 2 mm. NP dispersions were diluted with ultrapure water to a concentration of 0.1 mg mL^−1^. Afterward, a drop of NP suspension was deposited on a flat, mirror-polished silicon wafer mounted on an aluminium stub and dried at room temperature. Once the samples were dried, they were sputter-coated (Polaron E5100, Polaron Equipment, Watford, Hertfordshire, UK) with 2 nm of Pt/Pd (80:20) alloy to provide electrical conductance. Thermogravimetric analysis (TGA) was used to quantify the content of PAANa in the samples. TGA analyses were performed using a STA 449C Jupiter (Netzsch GmbH, Selb, Germany) apparatus. An amount of 10 mg ca. of sample was weighted in an alumina crucible and then heated from room temperature to 1200 °C under air flow with a heating rate of 10 °C/min. The chemical composition of dry samples was determined using an inductively coupled plasma optical emission spectrometer (ICP-OES) (Agilent 5100, Agilent Technologies, Santa Clara, CA, USA). Before analysis, 10 mg of samples was dissolved in 50 mL of 2 wt.% HNO_3_ solution in triplicate. The Ca and P content of the samples were measured by their atomic emission at 422.673 nm and 213.618 nm, respectively.

NPs in suspension at a 1 mg mL^−1^ concentration and at pH 7 were analyzed through dynamic light scattering (DLS) to determine their hydrodynamic diameter distribution and electrophoretic mobility (ζ-potential). DLS and ζ-potential analyses were performed using a Zetasizer Nano ZSP instrument (Malvern Instruments, Malvern, UK). The hydrodynamic diameter distribution of the samples was measured using hydroxyapatite refractive index (1.63) and water refractive index (1.33) as working parameters for the samples and the solvent, respectively. Size results are reported as the Z-average of the hydrodynamic diameter distribution of the particles of three measurements at 25 °C of at least 10 runs. ζ-potentials were quantified as the electrophoretic mobility at 25 °C of three separate measurements (maximum 100 runs each) by laser Doppler velocimetry using a disposable electrophoretic cell (DTS1061, Malvern Ltd., Worcestershire, UK) with the same sample and solvent parameters.

### 2.6. Biological Evaluation

HL-1 cardiac cells were grown in Claycomb medium (Sigma-Aldrich, St. Louis, MO, USA) supplemented with 10% fetal bovine serum (FBS) (Sigma-Aldrich, St. Louis, MO, USA), 1% penicillin–streptomycin (Pen-Strep 10,000 U/mi, Lonza, Basel, CH), 1% ultraglutamine (200 mM, Lonza, Basel, CH), and 0.1 mM of norepinephrine (Sigma-Aldrich, St. Louis, MO, USA) in gelatin/fibronectin-precoated T75 flasks (1 h, 37 °C). At full confluence, cells were split according to Claycomb’s instructions [23].

Intracellular calcium flux was measured using a Fluo-4 Direct™ Calcium Assay (Thermo Fisher Scientific, Waltham, MA, USA). The experiment was carried out over three days. On day 1, 10,000 HL-1 cells/well were plated in a black 96-well plate with 100 µL of complete medium. On day 2, treatment was added in Claycomb medium (with serum, starvation or starvation + NPs or MP) for 24 h. On day 3, cells were pre-treated with inhibitors of calcium fluxes from the sarcoplasmic reticulum, Thapsigargin 10 µM (Bio-Techne, Minneapolis, MN, USA), and Ryanodin 50 µM (Bio-Techne, Minneapolis, MN, USA) and then incubated with Fluo-4 Direct calcium reagent (Thermo Fisher Scientific, Waltham, MA, USA), prepared following the manufacturer’s instruction. After 1h of incubation at 37 °C, cells were stimulated with the LTCC-specific agonist Bay K8644 (Sigma-Aldrich, St. Louis, MO, USA) at the final concentration of 1 µM and calcium fluorescence signals (Ex 494 nm/Em 516 nm) detected for 30 min using a Synergy™ H4 Hybrid Multi-Mode Microplate Reader (BioTek Instrument, Winooski, VT, USA). Basal conditions (complete medium) and self-internalized MP (R7W-MP) were used as positive controls, while unloaded CaP NPs were used as negative controls. Both non-treated (NT) and all treated samples were subjected to serum starvation, a stress condition known to negatively affect LTCC density at the plasma membrane [14]. MP activity is expressed as intracellular calcium flux fold change in NT.

## 3. Results and Discussion

### 3.1. MP CaP NPs Characterization

CaP NPs functionalized with MP were prepared by simple one-pot wet precipitation. Aqueous calcium and phosphate precursors were co-precipitated in the presence of MP, following a pre-interaction step between the peptide and the calcium phase, as MP has a slightly negative net charge (−0.2 mV) at physiological pH. MP CaP NPs were synthesized at three MP nominal concentrations (50, 100, and 200 ppm) and compared to unloaded CaP NPs. The hydrodynamic diameter, polydispersity index, surface charge, NP concentration, Ca/P molar ratio, and drug loading of the samples are reported in Table 1.

Size and surface charge analyses show that CaP NPs exhibit a small hydrodynamic diameter of ca. 70 nm and a negative ζ-potential. The introduction of MP at 50 ppm during precipitation affects the NP properties, increasing the size and ζ-potential towards less negative values. Interestingly, further increases in MP concentration led to a decrease in ζ-potential towards more negative values, which was accompanied by a reduction in the average hydrodynamic diameter probably due to disaggregation induced by increased electrostatic repulsion between NPs. MP loading values were directly proportional to the nominal MP concentration, while NP concentration and the Ca/P ratio remained relatively unaffected. The Ca/P ratio of the samples is lower than the stoichiometric value of hydroxyapatite (HA), i.e., 1.67, indicating the formation of calcium-deficient HA. This Ca/P ratio is comparable to that of biogenic apatite found in bone tissue [24,25], indicating the biomimetic nature of MP CaP NPs.

**Figure 1:**
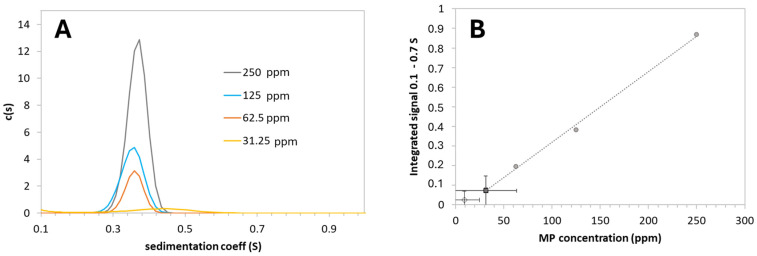
(**A**) Sedimentation coefficient distribution of MP at various concentrations (31.25–250 ppm) in water; (**B**) linear calibration curve (grey circles and dotted line) generated by integrating c(s) peak intensities corresponding to various MP concentrations and the values observed for the samples. Full rectangle: PAA MP CaP NPs; open diamond: MP CaP NPs. Error bars represent standard deviations from three repetitions.

Among the different MP CaP NPs, the sample prepared with the 200 ppm nominal MP concentration was selected for its superior peptide loading. This was measured using AUC to quantify any loosely bound MP fraction relative to the total loaded peptide amount. AUC is an analytic technique that effectively estimates the free drug fraction in nanomedicine products [26]. In our AUC experiments, CaP NPs sedimented much faster than non-bound MP, reaching the bottom of the measurement cell quickly; consequently, after the acceleration phase, only the sedimentation of the free MP could be observed (Figure S1, Supplementary Materials). The sedimentation coefficient distribution peak of non-bound MP appeared between 0.3 and 0.5 S in the calibration series, which aligns well with the molecular mass of the peptide (1326 kDa) (Figure 1A). The integrated signal intensity showed a linear correlation with concentration across the 31.25–250 ppm range (Figure 1B). In the 200 ppm MP CaP NP suspension, the concentration of non-bound MP fell below the lowest calibration point (31.25 ppm) (Figure 1B). Therefore, aliquots of the supernatant were carefully collected and analyzed by HPLC. The resulting non-bound MP concentration was 28.7 ± 2.1 ppm, corresponding to a fraction of ca. 30% of non-bound MP in the formulation, as reported in Table 2.

Therefore, the AUC data indicate that the majority of the loaded MP is retained by the NPs.

**Figure 2:**
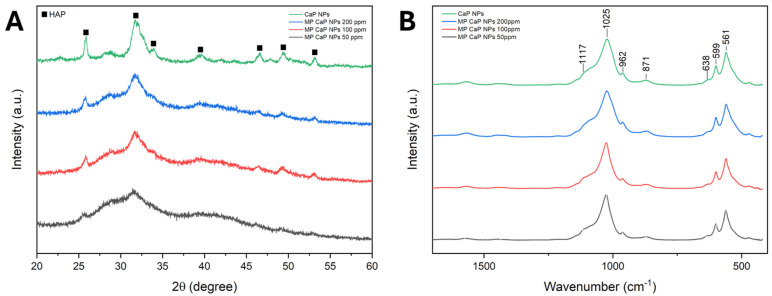
(**A**) PXRD patterns and (**B**) FTIR spectra of CaP NPs and MP-loaded CaP NPs.

The PXRD patterns, reported in Figure 2A, reveal that CaP NPs consist of an HA phase with low crystallinity, as evidenced by the broad and weak peaks in the diffractogram. The PXRD data align with the Ca/P ratio obtained, as calcium-deficient HA typically exhibits a lower Ca/P ratio compared to stoichiometric HA and is characterized by a low crystallinity degree, a feature consistent across different synthesis methods [27,28]. The addition of MP reduces the crystallinity of CaP NPs, likely due to the peptide’s role as a crystallization inhibitor. Peptides can recognize and bind to specific facets of the growing nanocrystals, thus regulating the size, shape, and crystallinity of the nanomaterial [29]. Specifically, depending on their sequence, length, and the position of certain amino acids, peptides may bind preferentially to specific facets of the crystal’s surface, preventing further growth in those regions [30].

The FTIR spectra of the four samples are shown in Figure 2B. Each sample exhibits the characteristic absorption bands of HA, corroborating the PXRD identification of the apatitic phase. This includes the main broad band centred at 1025 cm^−1^, with a shoulder at 1117 cm^−1^ corresponding to the triply degenerated antisymmetric stretching mode of apatitic PO_4_ groups (ν_3_PO_4_). Additional vibrational bands for these groups appear at 962 cm^−1^ (symmetric stretching mode, ν_1_PO_4_) and at 599 and 561 cm^−1^ (triply degenerated bending mode, ν_4_PO_4_) [31]. A small shoulder at 638 cm^−1^, indicative of the librational motion of apatitic hydroxyl ions (ν_L_OH), can be observed for all samples, and confirms the presence of HA [32]. Moreover, all samples present a small band at 871 cm^−1^, which can be attributed to the ν_2_CO_3_ vibration of the carbonate ion in an apatitic environment substituting phosphate ions (B-type substitution) [33]. The presence of carbonate ions likely results from the incorporation of the CO_2_ dissolved in the reaction solvent during precipitation. Additionally, two weak signals in the region between 1600 and 1400 cm^−1^ are observed, which can be attributed to the vibrational motions of absorbed acetate ions. Finally, no signals attributable to MP are present, most likely due to the low amount of peptide in the NPs. The complete FTIR spectra are reported in Figure S2 of the Supplementary Materials.

**Figure 3:**
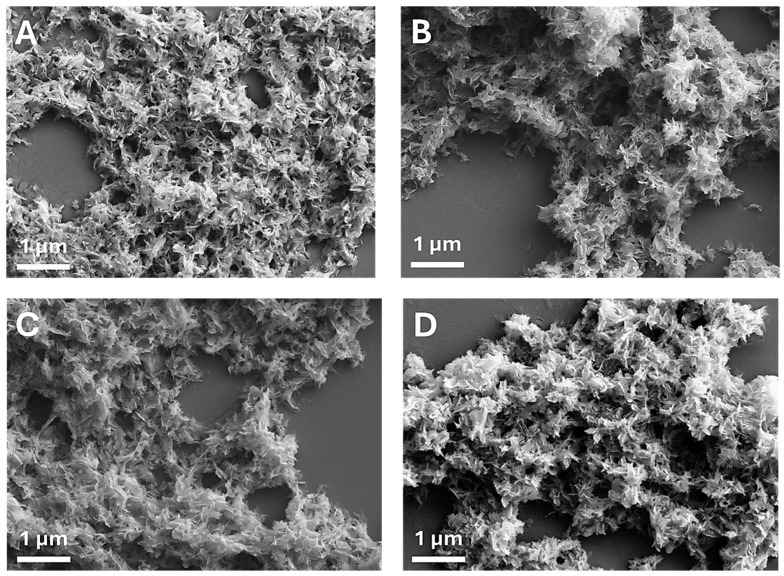
SEM micrographs of (**A**) CaP NPs, (**B**) MP CaP NPs 50 ppm, (**C**) MP CaP NPs 100 ppm, and (**D**) MP CaP NPs 200 ppm.

**Figure 4:**
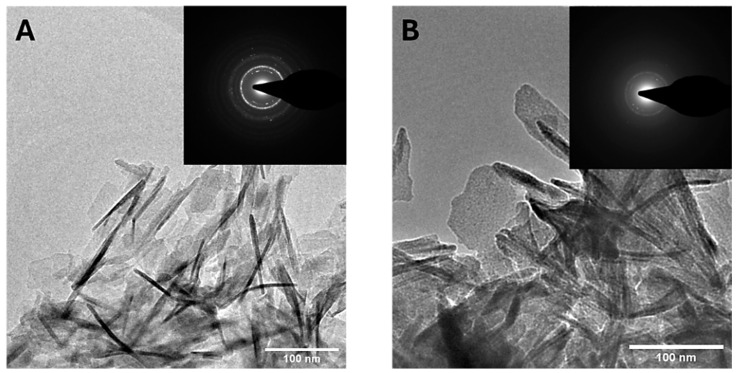
TEM micrographs of (**A**) CaP NPs and (**B**) MP CaP NPs 200 ppm. Insets: SAED patterns.

Figure 3 presents FEG-SEM micrographs of CaP NPs and MP CaP NPs. All samples exhibit a platelet-like morphology, which appears to be unaffected by MP concentration. The morphology of CaP NPs and MP CaP NPs 200 ppm was further examined by TEM (Figure 4). Both samples consist of elongated, flat platelets with dimensions along the major axis of ca. 100–150 nm. SAED patterns display randomly dotted rings in both samples, indicating that the NPs are polycrystalline. Furthermore, the SAED pattern for MP CaP NPs at 200 ppm show more diffusive borders, suggesting a lower degree of crystallinity compared to the control sample, consistent with PXRD data and supporting the fact that MP reduces the crystallinity of HA NPs. Figure S3 of the Supplementary Materials shows representative HR-TEM images of the platelet-like CaP NPs and MP CaP NPs, which display lattices planes with spacing of 0.80 and 0.34 nm corresponding to the d-spacing of the (100) and (002) planes of hexagonal phase of HA, respectively [34].

**Figure 5:**
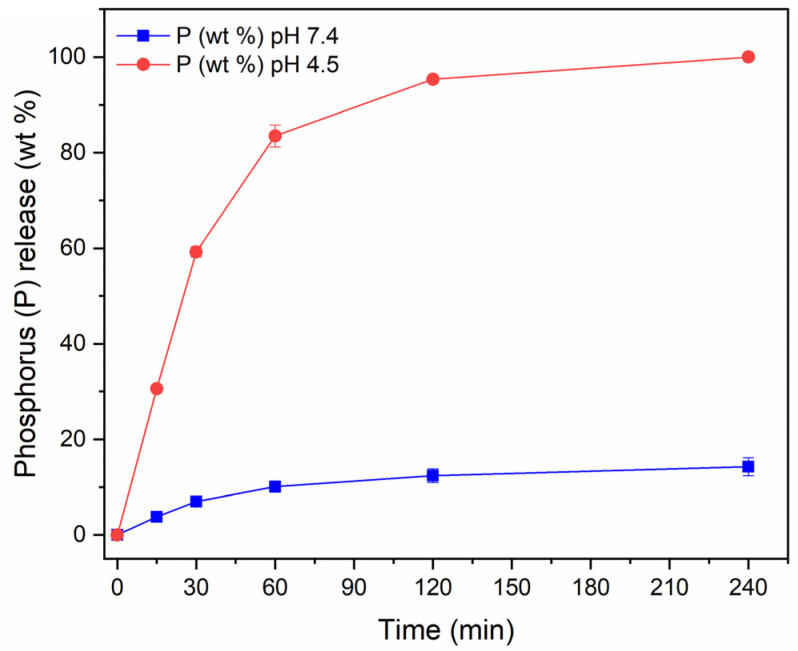
Release of phosphorus from MP CaP NPs 200 ppm in HEPES (blue) and acetate (red) buffers.

To gain further insight into the properties of the produced NPs, we evaluated the degradation of CaP NPs in HEPES and acetate buffers, by measuring phosphorus release (Figure 5). The data show that at physiological pH only approximately 10% of CaP NPs degrade within 4 h of incubation, whereas at acidic pH, the CaP NPs dissolve completely in the same time range. The pH-dependent solubility is indeed a well-known characteristic of CaPs and has been exploited to generate pH-triggered drug delivery systems [35,36,37,38].

### 3.2. MP CaP NPs 200 ppm Biological Evaluation

**Figure 6:**
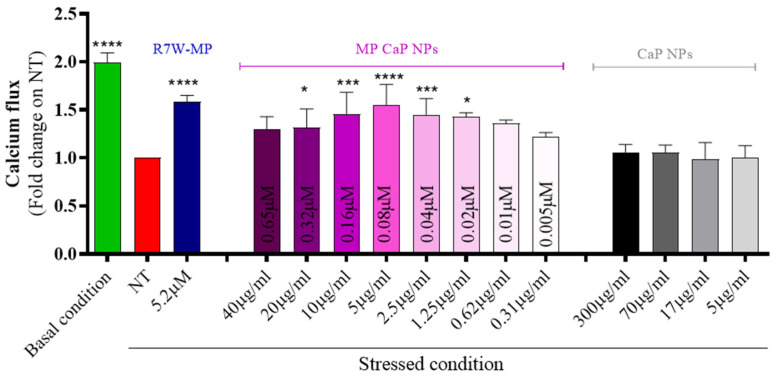
In vitro efficacy of MP CaP NPs 200 ppm on cardiac cells expressed as calcium flux. The treatment dose for CaP NPs is expressed as NP concentration (µg/mL), as indicated below the bars; the treatment dose for MP CaP NPs is expressed both as NP concentration (µg/mL) and MP concentration (µM), as indicated on the bars. Asterisks indicate levels of significance with respect to NT (Kruskal-Wallis test followed by Dunn’s multiple-comparison test): * *p* < 0.05, *** *p* < 0.001, **** *p* < 0.0001.

To assess the efficiency of MP CaP NPs in delivering and releasing the active therapeutic peptide, we conducted a series of in vitro analyses using HL-1 cardiac cells. Initially, MP CaP NPs were confirmed to be biocompatible upon exposure to HL-1 cells (Figure S4, Supplementary Materials). As MP has previously been shown to restore LTCC protein density at the plasma membrane and thus LTCC-dependent calcium fluxes under diseased or stressed conditions [14,39], we next performed an in vitro fluorescence-based functional assay to evaluate the effect of the formulation on the LTCC-dependent intracellular calcium fluxes (Figure 6). Serum-starved HL-1 cells were used to simulate stress conditions (i.e., LTCC dysregulation) and were exposed to incremental doses of MP CaP NPs. LTCC-dependent intracellular calcium fluxes in live cells were then monitored over time following stimulation with an LTCC-specific agonist (BAYK8644). As expected, a significant decrease in intracellular LTCC-specific calcium accumulation was observed under stressed conditions (e.g., NT and serum starvation) compared to the basal condition. Conversely, treatment with increasing doses of MP-loaded CaP NPs resulted in a progressive recovery of LTCC-dependent intracellular calcium accumulation, with a maximal effect observed around 5 µg/mL of NPs, corresponding to a peptide concentration of 0.08 µM. This effect was not obtained with unloaded CaP NPs, confirming that the therapeutic effect is due to the delivered MP. Notably, the naked MP, which is membrane impermeable, would not be effective without a carrier to reach its intracellular target and a self-penetrating version of MP containing the cell internalizing R7W sequence (R7W-MP) was therefore used as a positive control.

### 3.3. PAA MP CaP NP Characterization

The physico-chemical properties of MP-loaded CaP NPs functionalized with PAA (PAA MP CaP NPs) were compared with the new control NPs prepared in the absence of MP or PAA, which are referred to as PAA CaP NPs and MP CaP NPs, respectively. PAANa was chosen as it is a biocompatible and non-toxic polymer rich in carboxyl groups, which readily interact with the forming mineral phase [40,41]. The hydrodynamic diameter, polydispersity index, surface charge, PAA content, CaP NP concentration, and Ca/P molar ratio of the three samples are reported in Table 3.

**Figure 7:**
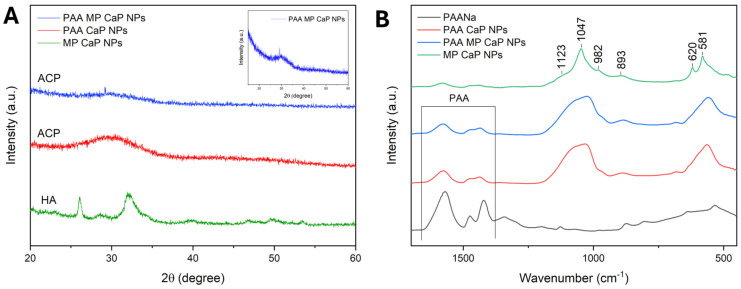
(**A**) PXRD patterns of PAA MP CaP, PAA CaP, and MP CaP NPs; (**B**) FTIR spectra of MP CaP, PAA MP CaP, and PAA CaP NPs and PAA. Inset 7A: PXRD pattern of PAA MP CaP NPs only.

Analysis of size and surface charge indicates that MP CaP NPs are characterized by a micrometric size and a slightly negative ζ-potential. Such a high size value could be explained by the limitation of DLS for polydisperse samples. The presence of micrometric particles can affect the accuracy of size distribution measurements, as DLS is sensitive to larger particles due to the sixth-power relationship between scattered intensity and particle radius. This sensitivity can result in inaccuracies when micrometric particles dominate the scattering signal in polydisperse samples [42]. Interestingly, following the addition of PAA to the reaction mixture, a sharp decrease in size down to 190 nm was observed, accompanied by a shift in the ζ-potential towards more negative values (−20 mV). These changes are attributed to the stabilizing effect of PAA, which adsorbs onto CaP NPs during nanocrystal growth, reducing the aggregation of the NPs by providing stabilization through electrostatic repulsion [43]. Moreover, PAA adsorption inhibited NP formation and growth, as evidenced by the lower concentration of NPs in the PAA MP CaP and PAA CaP NP samples compared to MP CaP NPs. The observed Ca/P ratios of 1.4–1.5 are typical of amorphous tricalcium phosphate (ATCP), the most common form of amorphous calcium phosphate (ACP), as previously reported in the literature [44] and consistent with the recorded PXRD patterns (Figure 7A). The presence of PAA increases the Ca/P compared to MP CaP NPs, likely due to the electrostatic interaction between the anionic polymer and Ca^2+^ ions. Furthermore, the coexistence of PAA and MP further elevates the Ca/P value, as observed when comparing PAA CaP and PAA MP CaP NPs. The presence of PAA also enhances MP loading, as evidenced by the comparison of MP CaP and PAA MP CaP NPs. This can be explained by the electrostatic interactions between the carboxyl groups of PAA and the peptide, which facilitate the retention of more MP in the NPs.

For PAA MP CaP NPs, the unbound MP fraction was quantified by AUC (Table 4). The concentration of non-bound MP fell below the lowest calibration point (31.25 ppm), as shown in Figure 1B. The resulting concentration of unbound MP in the supernatant was 8.8 ± 0.2 ppm, corresponding to approximately 23% of unbound MP.

Therefore, also in the case of PAA MP CaP NPs the majority of the loaded peptide is retained by the NPs.

The PXRD patterns reported in Figure 7A indicate that MP CaP NPs are composed of a pure HA phase with a low degree of crystallinity, while PAA-functionalized NPs appear amorphous, as evidenced by a single broad band centred at ca. 30° 2θ in the PXRD pattern, which is characteristic of ACP [33,45].

The PXRD results align with the FTIR spectra of the samples presented in Figure 7B. The amorphous nature of PAA MP CaP NPs and PAA CaP NPs is indicated by the presence of broad and non-resolved phosphate vibrational bands. In addition, the ν_3_PO_4_, ν_1_PO_4_, and ν_4_PO_4_ bands of these samples are shifted to 1024, 963, and 560 cm^−1^ respectively, which are approximately 20 cm^−1^ lower than those of MP CaP NPs. This downshift has been previously reported for the FTIR spectra of ACP, compared to HA [44]. The bands in the region between 1600 and 1400 cm^−1^ are more pronounced in the PAA samples than in MP CaP NPs and are attributed to the carboxyl groups of PAA, suggesting an association between the polymer and the NPs. The complete FTIR spectra are available in Figure S5 of the Supplementary Materials. Overall, compositional, PXRD, and FTIR analyses indicate that the presence of PAA inhibits CaP NP crystallization, likely due to PAA adsorption onto newly formed amorphous particles, which stabilizes them and prevents their crystallization.

**Figure 8:**
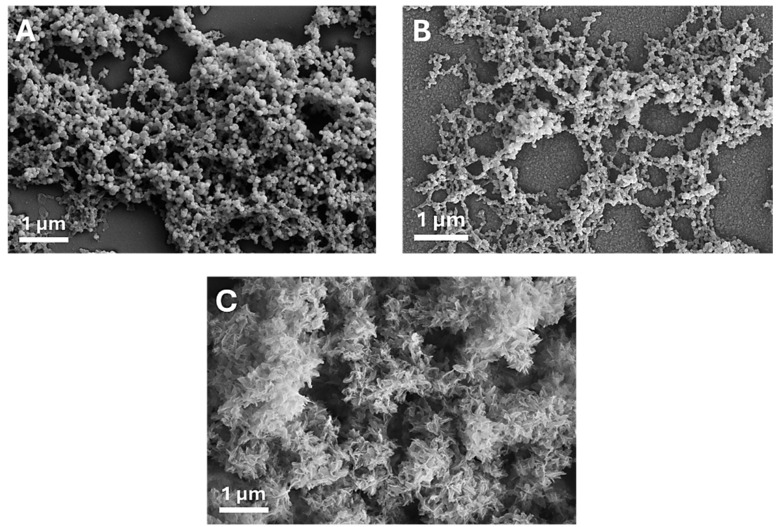
SEM micrographs of (**A**) PAA MP CaP NPs, (**B**) PAA CaP NPs, and (**C**) MP CaP NPs.

Figure 8 presents the FEG-SEM micrographs of PAA MP CaP NPs and the control samples. PAA-free MP CaP NPs consist of platelet-like NPs comparable to the ones reported above. In contrast, the presence of PAA results in the formation of smaller and round-shaped NPs with a diameter of approximately 100 nm. This morphological change is likely attributable to the stabilization of the amorphous phase by PAA. In fact, amorphous particles typically lack a preferred growth direction, forming rounded particles, as driven by surface energy minimization [46].

**Figure 9:**
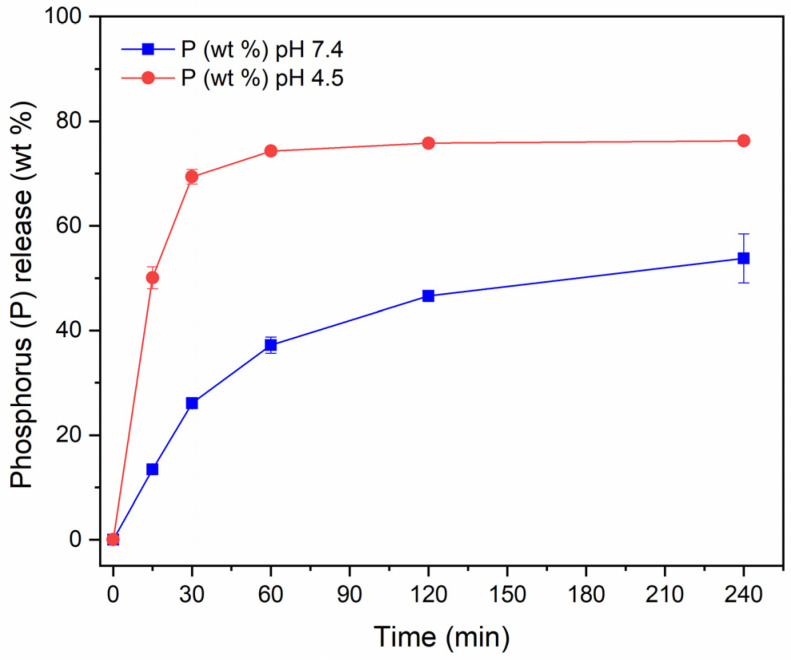
Release of phosphorus from PAA MP CaP NPs in HEPES (blue) and acetate (red) buffers.

The degradation of PAA MP CaP NPs in HEPES and acetate buffer was also investigated (Figure 9). The degradation curves clearly show that PAA MP CaP NPs are rapidly degraded even at physiological pH. Specifically, approximately 50% of the NPs are degraded after 2 h, in contrast to MP CaP NPs 200 ppm, where only about 10% of the NPs dissolve in the same time frame. This increase in the degradation rate could be attributed to the amorphous nature of the NPs, which are more soluble than their crystalline counterparts, leading to their rapid degradation [47].

### 3.4. PAA MP CaP NPs Biological Evaluation

**Figure 10:**
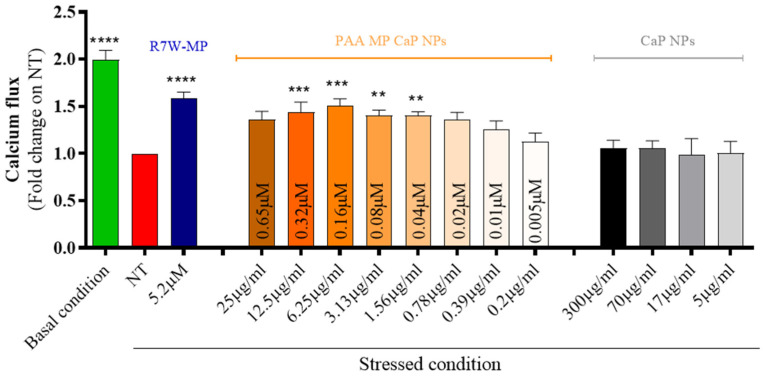
In vitro efficacy of PAA MP CaP NPs on cardiac cells expressed as calcium flux. The treatment dose for CaP NPs is expressed as NP concentration (µg/mL), as indicated below the bars; the treatment dose for PAA MP CaP NPs is expressed both as NP concentration (µg/mL) and MP concentration (µM), as indicated on the bars. Asterisks indicate levels of significance with respect to NT (Kruskal-Wallis test followed by Dunn’s multiple-comparison test): ** *p* < 0.01, *** *p* < 0.001, **** *p* < 0.0001.

As reported above for MP CaP NPs, PAA MP CaP NPs also exhibited good biocompatibility towards cardiomyocytes (Figure S4, Supplementary Materials). To evaluate their efficiency in delivering and releasing the active therapeutic peptide within cardiac cells, we conducted an in vitro fluorescence-based functional assay in HL-1 cells, as described for MP CaP NPs (Figure 10). Treatment with increasing doses of PAA MP CaP NPs restored LTCC-dependent intracellular calcium fluxes in stressed conditions, with the maximum effect observed at an NP concentration of 6.25 µg/mL, corresponding to 0.16 µM of delivered MP. The need for a higher concentration to achieve the peak of calcium fluxes may be due to the larger size of PAA MP CaP NPs (182 ± 4 nm, Table 3) compared to MP CaP NPs (71 ± 1 nm, Table 1) and thus to the different mechanism of endocytosis, where slower macropinocytosis may occur for the larger particles [48].

In both cases, CaP NPs were synthesized using a biomineralization-inspired approach and loaded with a cardio-specific therapeutic peptide. Both systems retained the majority of the loaded peptide and restored the calcium flux in vitro. NPs prepared with PAA exhibited a small size of less than 100 nm and a reduced degradation rate due to their crystalline nature, whereas those synthesized in the presence of PAA had a larger size of about 200 nm and a faster degradation rate in line with their amorphous character. These differences suggest that the two nano-systems could serve distinct applications in the biomedical field, depending on specific requirements.

## 4. Conclusions

This study presents novel peptide-loaded CaP NPs as drug delivery systems aimed at restoring cardiac function. Our results demonstrate that CaP NPs can be loaded with a cardio-specific therapeutic peptide via a biomineralization-inspired approach and effectively retain the majority of the loaded therapeutic molecule. The crystallinity, size, surface charge, and morphology of the loaded-NPs can be finely tuned through variation of synthesis conditions. In vitro tests on cardiac cells demostrated that both MP CaP and PAA MP CaP NPs are biocompatible and effective in restoring the intracellular calcium flux under stressed conditions, highlighting their ability to deliver and release the therapeutic MP peptide. However, whether the MP peptide, used here as a reference drug for designing novel NPs, directly influences other calcium-handling mechanisms remains to be investigated in future studies. Further investigations, including in vivo testing in appropriate animal models of heart failure, are needed to confirm these promising results. Overall, this work paves the way for further formulation optimization and opens up new perspectives for CaP NPs as viable nanomaterials for CVD treatment.

## Figures and Tables

**Table 1:** Physico-chemical characterization of CaP NPs and MP CaP NPs.

Sample	Z-Average (nm)	PDI	ζ-Potential (mV)	CaP NP Concentration (mg/mL)	Ca/P (mol)	MP Loading (wt.%)
CaP NPs	70 ± 1	0.19	−9.4 ± 0.6	3.48 ± 0.08	1.48	-
MP CaP NPs 50 ppm	96 ± 2	0.18	−1.7 ± 0.7	3.38 ± 0.01	1.45	0.4
MP CaP NPs 100 ppm	85 ± 1	0.27	−2.1 ± 0.6	3.29 ± 0.04	1.46	1.0
MP CaP NPs 200 ppm	71 ± 1	0.21	−6.0 ± 1.0	3.78 ± 0.04	1.46	2.4

**Table 2:** Free and retained MP in MP CaP NPs 200 ppm.

Sample	MP Loading (wt.%)	Free MP (%)	Retained MP * (%)	AUC-Corrected MP Loading (wt.%)
MP CaP NPs 200 ppm	2.4	30 ± 2	70 ± 2	1.7

* Calculated as the difference with respect to the percentage of free MP.

**Table 3:** Physico-chemical characterization of PAA MP CaP, PAA CaP, and MP CaP NPs.

Sample	Z-Average (nm)	PDI	ζ-Potential (mV)	CaP NPs Concentration (mg/mL)	Ca/P (mol)	PAANa (wt.%)	MP Loading (wt.%)
PAA MP CaP NPs	182 ± 4	0.14	−20.0 ± 1.0	1.23 ± 0.01	1.51	13	3.2
PAA CaP NPs	189 ± 2	0.12	−18.5 ± 0.4	1.40 ± 0.01	1.40	13	-
MP CaP NPs	2505 ± 289	0.39	−4.0 ± 0.6	3.11 ± 0.05	1.35	-	1.3

**Table 4:** Unretained and retained MP in PAA MP CaP NPs.

Sample	MP Loading (wt.%)	Free MP (%)	Retained MP * (%)	AUC-Corrected MP Loading (wt.%)
PAA MP CaP NPs	3.2	23 ± 1	77 ± 1	2.5

* Calculated as the difference with respect to the percentage of free MP.

## Data Availability

The data presented in this study are available on request from the corresponding author.

## Supplementary Materials

The following supporting information can be downloaded at https://www.mdpi.com/article/10.3390/nano15020094/s1: Figure S1: Typical sedimentation profile (interference-based signal at various time points from dark blue to red), residuals, and fit results for a sample containing nanoparticles and a very low concentration of free peptide. Figure S2: FTIR spectra of CaP and MP-loaded CaP NPs. Figure S3: (1) SAED pattern and (2) HR-TEM images of (A) CaP NPs and (B) MP CaP NPs 200 ppm. Figure S4: Viability of HL-1 cells after exposure to MP CaP NPs and PAA MP CaP NPs at increasing concentrations. “Control” refers to cells without any treatment. Figure S5: FTIR spectra of MP CaP NPs, PAA-functionalized CaP NPs, and PAANa.
