# Supplementary material for: Calcium Phosphate Nanoparticles Functionalized with a Cardio-Specific Peptide
Source: Nanomaterials (Basel). 2025 Jan 9;15(2):94. doi: 10.3390/nano15020094 (PMC11767714; doi:10.3390/nano15020094)
Supplement: Supplementary file 1 [file nanomaterials-15-00094-s001.zip › nanomaterials-3352441-supplementary.pdf]

Supplementary Information

Calcium Phosphate Nanoparticles Functionalized with a Cardio-specific Peptide

Federica Mancini[1], Lorenzo Degli Esposti[1], Jessica Modica[2,3], Dora Mehn[4], Otmar Geiss[4], Daniele Catalucci[2,3], Alessio Adamiano[1], Michele Iafisco[1,*]

[1]Institute of Science, Technology and Sustainability for Ceramics (ISSMC), National Research Council (CNR), Faenza (RA), Italy.

[2]IRCCS Humanitas Research Hospital, Humanitas Cardio Center, Rozzano (MI), Italy.

[3]Institute of Genetic and Biomedical Research (IRGB) – Milan unit, National Research Council (CNR), Milan, Italy.

[4]European Commission, Joint Research Center (JRC), Ispra (VA), Italy.

*Correspondence should be addressed to Michele Iafisco

E-mail: michele.iafisco@issmc.cnr.it

Tel: +39 0546699730

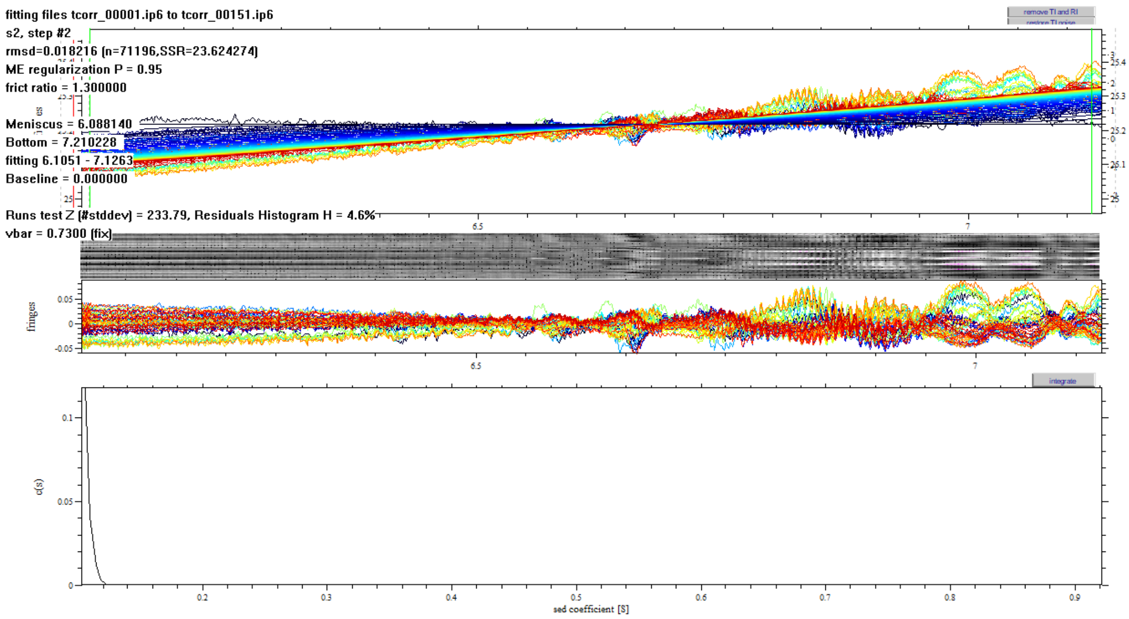

Figure S1. Typical sedimentation profile (interference-based signal at various time points from dark blue to red), residuals and fit results for a sample containing nanoparticles and a very low concentration of free MP peptide.

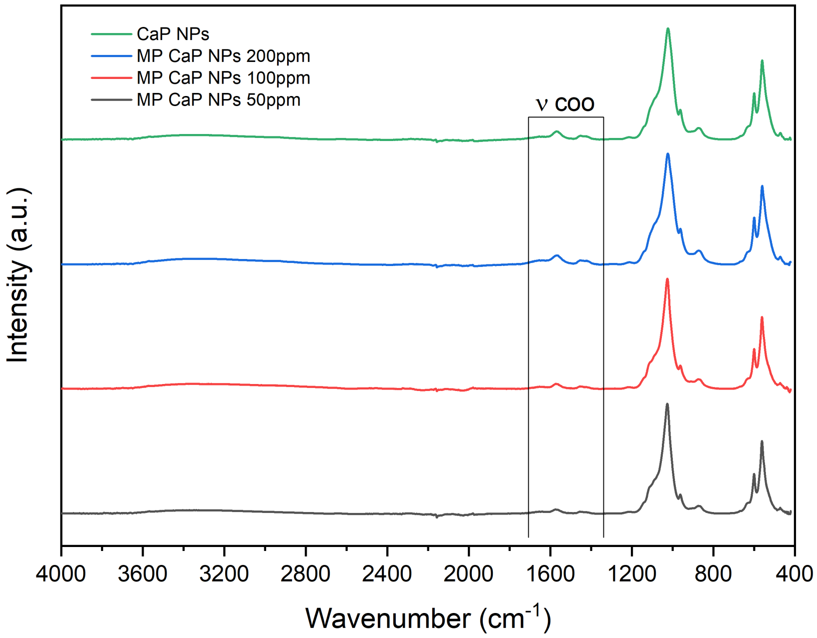

Figure S2. FTIR spectra of CaP NPs and MP-loaded CaP NPs.

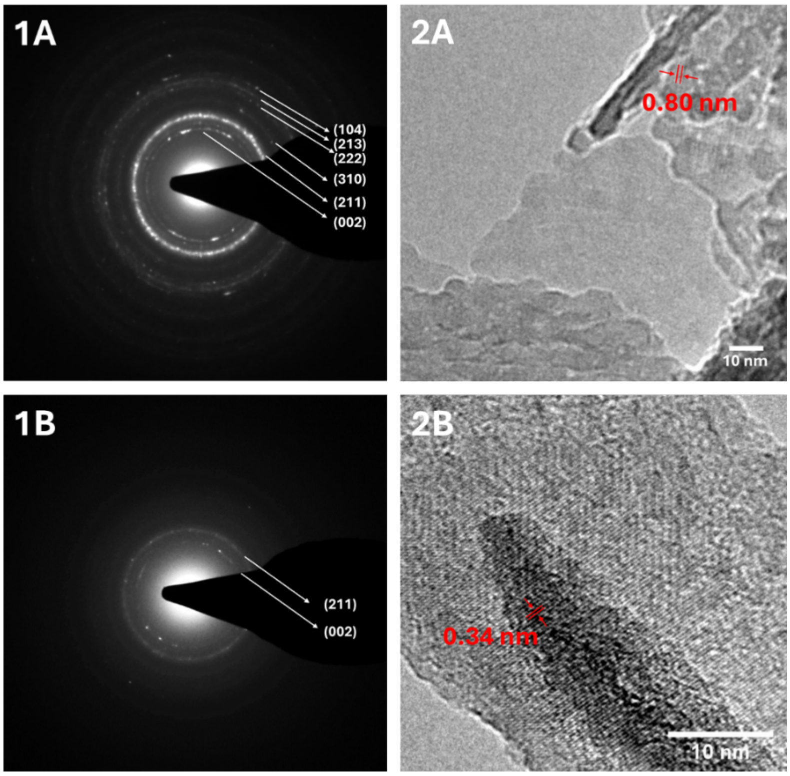

Figure S3. (1) SAED pattern and (2) HR-TEM images of (A) CaP NPs and (B) MP CaP NPs 200ppm.

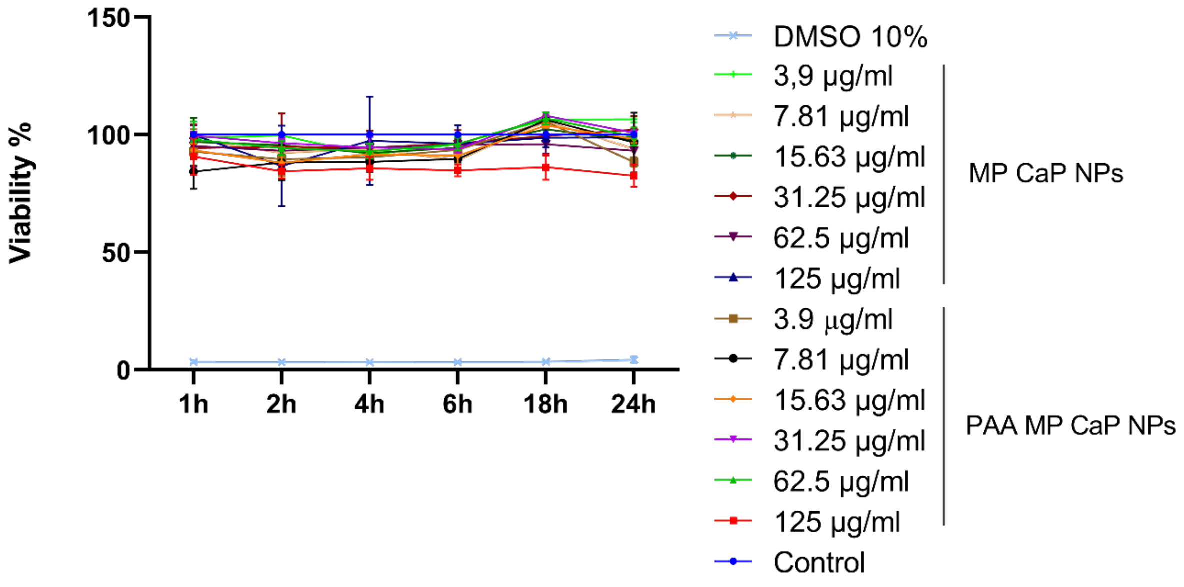

Figure S4. Viability of HL-1 cells after exposure to MP CaP NPs and PAA MP CaP NPs at increasing concentrations. "Control" refers to cells without any treatments.

Note to Figure S4

The effect of NPs on HL-1 cell viability was analysed using RealTime-Glo^{TM} MT Cell Viability Assay kit (Promega S.r.l, Milan, Italy) following the manufacturer's instructions. Briefly, 10,000 cells/well were seeded in white-walled 96-well plates. After 24 hours, HL-1 cells were treated with increasing concentrations of MP CaP NPs or PAA MP CaP NPs. DMSO (10%) was used as positive control. To continuously monitor cell viability in real time, RealTime-Glo reagents were added simultaneously with the NPs. Luminescence intensity, indicative of viable cells, was measured at the desired time points up to 24 hours using a GloMax® Discover Microplate Reader (Promega S.r.l, Milan, Italy).

To assess the potential cytotoxic effects of MP CaP NPs and PAA MP CaP NPs, HL-1 cardiac cells were treated with incremental doses of NPs (0–125 µg/ml), and viability was measured using the RealTime-Glo^{TM} MT Cell Viability Assay. As shown in Figure S4, both types of NPs were well tolerated by the cells, with more than 85% viability observed up to 125 µg/ml of NPs at 24 hours post-administration.

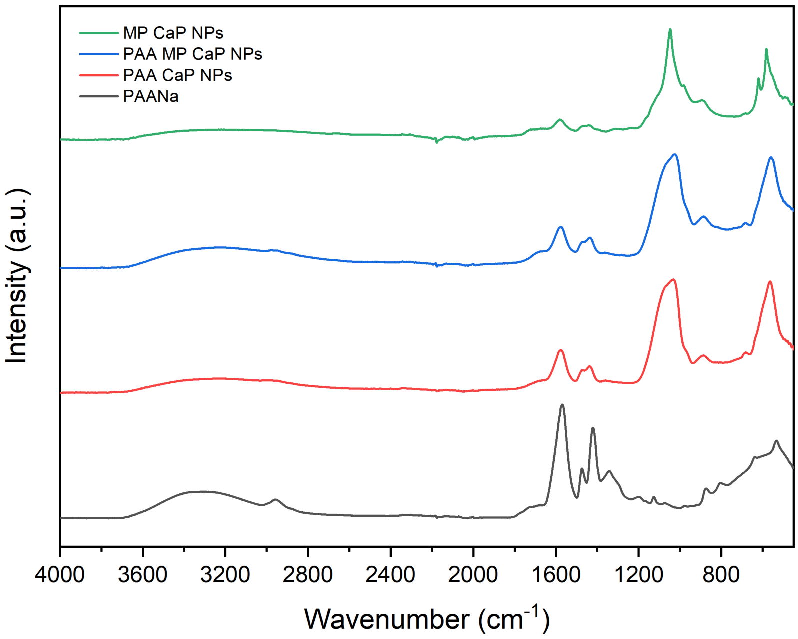

Figure S5. FTIR spectra of MP CaP NPs, PAA-functionalized CaP NPs and PAANa.
